# The effect of punishment on cooperation in a multilevel public goods game: compositional data analysis

**DOI:** 10.1038/s41598-026-39950-1

**Published:** 2026-03-02

**Authors:** Yoko Kitakaji, Misato Inaba

**Affiliations:** 1 Faculty of Economics, Kindai University, Higashi-osaka, Japan; 2 Research Institute for Socionetwork Strategies, Kansai University, Suita, Japan; 3 The Institute for Diversity and Inclusion, Hiroshima University, 1-1-1, Kagamiyama, Higashi-Hiroshima, Hiroshima Japan; 4 Graduate School of Humanities and Social Sciences, Hiroshima University, Higashi-Hiroshima, Japan

**Keywords:** Cooperation, Punishment, Multilevel public goods game, Sanction, Compositional data, Nested social dilemma, Human behaviour, Psychology

## Abstract

When addressing collective challenges, cooperation is essential both within and across subgroups. In multilevel social dilemmas, cooperation can occur at two levels: local cooperation (within one’s subgroup) and global cooperation (across subgroups). While punishment has been shown to foster local cooperation in standard, non-multilevel public goods games, its role in promoting global cooperation remains unclear. To examine cooperative behavior at both levels, the present study involves 120 participants and employs a multilevel public goods game with punishment. Participants decided how to allocate endowments among self, local, and global accounts. We tested (i) whether punishment promotes local and/or global cooperation, (ii) whether individuals punish in-group or out-group members more, and (iii) what behaviors are punished. Using compositional data analysis suitable for examining allocation ratios among multiple targets, we found that punishment promoted both local and global cooperation by primarily targeting non-cooperators. Importantly, punishment behavior toward in-group and out-group members did not differ. These findings suggest that punishment can facilitate cooperation beyond subgroup boundaries and provide a potential mechanism for sustaining collective action across groups.

**Supplementary Information:**

The online version contains supplementary material available at 10.1038/s41598-026-39950-1.

## Introduction

In the context of human societies, which have developed multilayered structures consisting of multiple local subgroups within a broader global collective, cooperation within and across subgroups is essential. In such multilevel social systems, cooperation can occur at two levels: local cooperation (within one’s subgroup) and global cooperation (across or between subgroups). Researchers have traditionally examined cooperation within a single group as a social dilemma—a conflict between individual and collective interests^1^. However, modern collective challenges, such as climate change and resource management, increasingly require global cooperation to transcend subgroup boundaries and benefit all members across groups. Although experimental evidence on global cooperation remains limited, especially compared to that of standard social dilemmas, recent studies provide important insights into the possibility of achieving such cooperation.

The multilevel public goods game (MPGG) is an experimental design examining cooperation in a nested, global–local group structure^2–4^. In an MPGG, individuals allocate resources between two types of public goods: local public goods (benefiting only their subgroup) and global public goods (benefiting all participants across subgroups). Two parameters determine the balance between local and global cooperation. The first parameter is the marginal per capita return (MPCR), which captures the individual return from contributing; the second parameter is the total benefit (TB), defined as TB = MPCR × n (with n being the number of recipients), capturing the collective return. The higher the global level of TB, the stronger the improvement of social welfare through global cooperation. On the other hand, when the MPCR of the global public goods is smaller than that of the local one, cooperation at the global level is more challenging. According to experimental evidence, increasing the global MPCR induces a leveling-up effect, with more allocations directed to the global public good^4–8^. However, this effect is often driven by substitution from the local public good, without increasing overall allocations^5,7,8^. At the same time, adding a new category of public good can crowd in additional allocations^9^. In modern societies, global cooperation is essential for addressing large-scale problems such as climate change, yet it remains individually less rewarding than local cooperation. To reproduce this tension experimentally, our MPGG design adopts a parametrization in which contributions to the global public good yield a higher TB for all but a lower MPCR for the contributor than local contributions. This configuration allows us to model the challenge of promoting global cooperation despite its higher social efficiency. In our experiment, allocating one point to the local yields MPCR_local_  = 2/3 to 3 members (TB_local_  = 2), whereas allocating one point to the global good yields MPCR_glob_  = 1/2 to 6 members (TB_glob_ = 3). Thus, private returns favor local over global, while TBs favor global over local.

In addition to these structural parameters, standard public goods games have highlighted several behavioral motives for contributing, such as altruism, reciprocity, inequality aversion, guilt aversion, and confusion (for a review, see^10^). Although this study does not consider multilevel settings, such motives may also play a role when participants decide how to allocate their resources among self, local, and global accounts.

Existing studies have highlighted the challenges of achieving global cooperation in MPGGs due to the presence of in-group favoritism^5,8,9,11,12^, which refers to a psycho-behavioral tendency to favor members of one’s own in-group over those from out-groups^13–15^. While some studies on MPGG have found more local than global cooperation^5,8,11,12^, others have shown the opposite tendency^16,17^. Studies manipulating MPCR and TB show that, even when the TB of the global good exceeds that of the local good, local allocations tend to persist and overall allocations remain unchanged, thereby reducing relative efficiency. However, invoking parochial identity does not necessarily weaken responsiveness to MPCR^6,8^. Moreover, subjects’ cooperation tendencies change depending on categorization, social identity, communication frames, and intergroup interactions^2,18–20^. While these studies have focused on allocation decisions, relatively few have examined punishment in multilevel settings^21^, despite punishment promoting cooperation in standard social dilemmas^22–25^.

To address this gap, we examine how punishment affects both local and global cooperation, with particular attention to its direction toward in-group and out-group members. Individuals punish others based on their beliefs about which cooperative norms should be prioritized—whether cooperation with the local group or the global group constitutes the appropriate standard of behavior. Accordingly, individuals who endorse a local cooperative norm may punish those who fail to cooperate with their local group, whereas individuals who adopt a global cooperative norm may punish those who do not contribute to the global collective. In turn, those subjected to punishment infer how they are expected to behave from the punishment they receive and can adjust their behavior accordingly.

In an MPGG, several distinct punishment dynamics can arise. First, non-cooperation—failing to cooperate with either the local or the global group—may be punished. Commonly observed in standard social dilemmas, this punishment type encourages contributions to both the local and global groups, thereby sustaining the coexistence of cooperation at both levels.

Second, failure to cooperate with the local group may be the primary target of punishment. Prior research has established that in-group cooperation is prioritized over out-group cooperation^26,27^ and that norm violations are sanctioned through costly punishment^25^. Importantly, punishment is stronger when directed at in-group members who fail to cooperate than at out-group defectors^28^, suggesting that local group cooperation is a primary focus of norm enforcement. Under such a norm, in-group members may punish not only non-cooperators but also those who prioritize global cooperation at the expense of their local group. Out-group members’ non-cooperation may go unpunished, while their behavior may be sanctioned because it damages the in-group. This dynamic supports parochial cooperation—that is, cooperation within the local group.

Third, punishment may focus on the failure to cooperate with the global group, reflecting a preference for maximizing collective interests. Under this norm, local cooperation undermining global welfare may be punished. Punishment directed at non-global cooperators can promote global cooperation; when such a norm is widely accepted, punishment practices converge between in-groups and out-groups.

Cooperation within one’s local group can be regarded as non-cooperation by out-groups as it does not benefit them. However, punishment motivated by such a self-serving criterion is unlikely to be sustained in the long term.

Importantly, these dynamics unfold not only in one-shot games but also in repeated interactions. In repeated MPGGs, as subjects observe and respond to others’ actions over time, their behaviors evolve; the stability of cooperative norms depends on these iterative adjustments. Thus, the emergence and persistence of particular punishment patterns cannot be fully understood without considering the dynamic nature of repeated interactions. Even when members initially differ—some engaging in global cooperation, others in parochial cooperation or non-cooperation—shared norms may gradually emerge through repeated punishment and learning. When a cooperative norm is widely supported, it may become established within the group, promoting stable cooperation at either local or global level. However, when multiple competing norms coexist—such as some individuals punishing local and others punishing global cooperation—the group may fail to converge on a single standard. In such cases, mutual punishment can escalate, ultimately discouraging cooperation at both levels and resulting in widespread non-cooperation.

Building on these considerations, Otten et al.^21^ provided one of the few empirical examinations of punishment in MPGGs and found that punishment failed to promote cooperation unless participants had prior experience with standard public goods games. They attribute this ineffectiveness to normative disagreement—the lack of consensus among group members about appropriate cooperation norms. MPGGs feature multiple plausible cooperation norms (local, global, or mixed contributions), creating ambiguity about which behavior punishment aims to enforce. This normative ambiguity prevented groups from coordinating and cooperating efficiently. We argue that such normative disagreement is likely to be particularly pronounced between local groups—an aspect not directly examined in Otten et al.’s study^21^. Therefore, differentiating between in-group and out-group punishment offers a more fine-grained approach to understanding its effects in MPGGs.

To address these issues, we designed an experiment using a MPGG in which participants allocated resources among global group, local group, and themselves. The game was implemented under two conditions: a control condition without punishment and a condition with punishment in which participants could sanction others after the allocation stage. Each game consisted of 20 repeated rounds. In the punishment condition, participants were endowed with points that could be used to punish other members. To ensure that sanctions imposed a meaningful deterrent cost, each point spent on punishment reduced the target’s payoff by three points. In line with prior studies on costly punishment^24,28^, the points used for punishment were not returned to the punisher, so that punishing others entailed a personal cost. Unused punishment points were added to the punisher’s own earnings so that participants were not disadvantaged simply by being allocated punishment resources. This design allowed us to examine the role of costly punishment in shaping cooperation, while maintaining consistency with established experimental paradigms.

Importantly, as contributions to the three accounts (global group, local group, self) necessarily sum to a constant, participants’ allocation decisions produced compositional data. This structure generates negative correlations between variables, making standard statistical approaches insufficient. To capture these dependencies, we employed compositional data analysis, which provides a more rigorous framework for evaluating cooperation in MPGGs (see Methods for details).

Based on this design and analytic approach, we addressed the following research questions: (i) Can punishment increase cooperation in MPGGs and, if so, does it primarily enhance local cooperation or global cooperation? (ii) Are in-group or out-group members more frequently subjected to punishment? and (iii) What specific behaviors tend to incur punishment?

## Results

### Allocation decisions

**Fig. 1:**
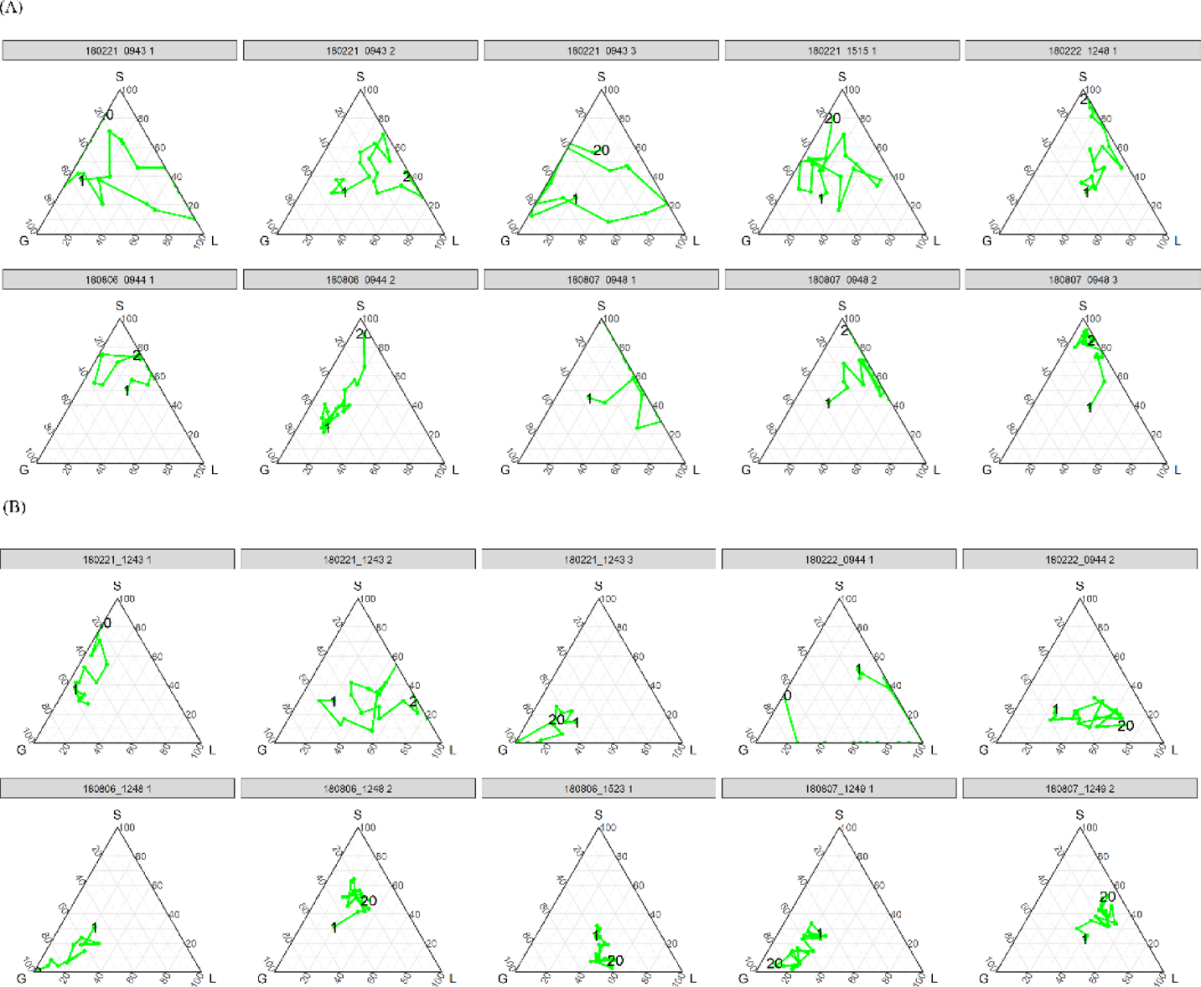
Transition of allocation mean for each group in the control condition (**A**) and the punishment condition (**B**). Each vertex in the ternary plot indicates an allocation type. The top vertex (S) is self, the right vertex (L) is local, and the left vertex (G) is global. The closer it is to the vertex, the greater the allocation to the account. The numbers on the line indicate the period numbers. In the control condition, there was a gradual shift toward non-cooperation over time, despite intermediate increases in cooperation with locals and global cooperation. In contrast, under the punishment condition, most groups demonstrated a preference for global or local cooperation over self-interest. Not all groups exhibited a mix of global and local cooperation; punishment induced global cooperation in some groups and local cooperation in others.

Figure 1 shows the allocation transitions in periods by group. In the control condition, groups were not readily classifiable as cooperative (local or global) or non-cooperative at the outset, but gradually shifted toward non-cooperation as the periods progressed. However, it did not always approach the vertex (self) directly; although the allocation to global decreased and that to local increased, the allocation to self eventually increased. Conversely, in the punishment condition, few groups went for self, while the majority headed for global and local cooperation. At group-level, the mix of global and local cooperation did not always occur. Punishment could lead to global cooperation in some groups and to local cooperation in others.

**Fig. 2:**
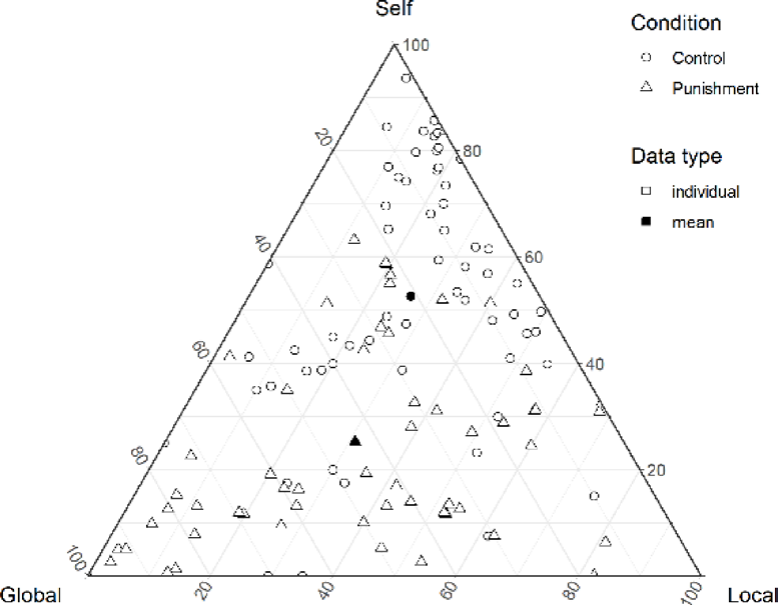
The mean allocation rate per period by condition. Each vertex indicates an allocation type. The closer it is to the vertex, the greater the allocation to the account. The circles denote the control condition. The triangles denote the punishment condition. Open symbols represent the data for each participant, and filled symbols indicate the mean of each condition. This figure illustrates the individual mean allocation rate. The mean allocation rate to self was the highest, followed by local and global in the control condition. In the punishment condition, the global allocation was the highest, followed by local and self.

Next, in the control condition, mean allocation rate per period to self (*M*_*self*_ = 52.6%) was the highest, followed by local (*M*_*local*_ = 26.4%) and global (*M*_*global*_ = 21.0%). In the punishment condition, global allocation was the highest (*M*_*global*_ = 43.8%), followed by local (*M*_*local*_ = 31.0%) and self (*M*_*self*_ = 25.2%) (Fig. 2).

**Fig. 3:**
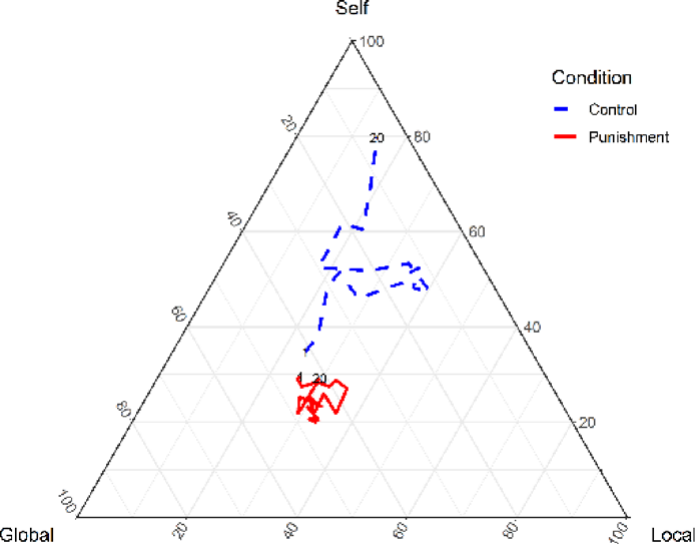
The transition of mean allocation rate by condition per period. The blue dashed line denotes the control condition. The red solid line denotes punishment condition. The numbers on the line indicate the period numbers. In the control condition, allocations fluctuated between global cooperation and local cooperation. Under the punishment condition, the allocation rates remained stable, with global allocation consistently highest. Despite this, some were still allocated to the self and local.

Figure 3 shows the transition of aggregate (mean) allocation proportions. In the control condition, allocations on average shifted from global to local and back to global. In the punishment condition, the mean proportions remained relatively stable, with global allocations remaining the highest across periods. However, as the group-level ternary trajectory plots in Fig. 1 show, paths diverge across groups: some display a net drift toward the global account, others drift toward the local account; several oscillate between the two, with occasional excursions toward self in early periods before returning to the two public accounts (local and global).

Hence, we present the results of the log-ratio analysis (see the Methods section for details). Before log-ratio transformation, zero values required imputation as the log ratio cannot be calculated otherwise. We replaced 0 with smaller values (0.025) according to Martín-Fernández, Barceló-Vidal, and Pawlowsky-Glahn’s method^30^. We employed Arai and Ohta’s^31^ R code for zero-replacement. We used an additive log-ratio transformation to calculate the local and global allocation log-ratios based on each participant’s self-allocation value with the alr function of the composition package in R.

A linear mixed model was fitted with *condition* (control vs. punishment; between-participants) and *allocation type* (local vs. global; within-participants) as fixed effects, and 6-person group, 3-person group, and individual as random effects (Table 1). Results are displayed in Table 2, with only the control conditions extracted. The analysis revealed a significant main effect of condition (*b* =  − 1.01, SE = 0.42, 95% CI [− 1.89, − 0.13], *t* =  − 2.37, *p* = 0.026) and a significant condition × allocation-type interaction (*b* =  − 0.98, SE = 0.30, 95% CI [− 1.58, − 0.38], *t* =  − 3.23, *p* = 0.002; Table 1), suggesting that, relative to the control condition, punishment increased allocations to both local and global accounts. Log-ratio analysis showed that allocation to local and global was greater in the punishment condition than in the control condition. In the latter, allocation to self was greater than that to the local group (*b* =  − 0.73, SE = 0.27, 95% CI [− 1.32, − 0.13], *p* = 0.02), and local allocation exceeded that to global (*b* =  − 0.59, SE = 0.20, 95% CI [− 0.99, − 0.19], *p* = 0.005). In contrast, under punishment, no significant difference was found between self and local allocations (*b* = 0.28, SE = 0.30, 95% CI [− 0.34, 0.90]) or between local and global allocations (*b* = 0.39, SE = 0.21, 95% CI [− 0.03, 0.82]). In summary, participants in the punishment condition displayed more cooperative allocation patterns than those in the control condition, contributing more to local and global public goods.

**Table 1:** Mixed-model analysis of cooperative behavior.

Random effects	Variance	*Std.Dev*
Individual	0.47	0.69
3-person group	0.59	0.77
6-person group	0.30	0.55
Residual	1.38	1.18

Fixed effects	Estimates	*SE*	*95% CI*	*t*	*p*
Intercept	0.28	0.30	− 0.34 to 0.90	0.94	0.357
GvsI	0.39	0.21	− 0.03 to 0.82	1.82	0.071
Control	− 1.01	0.42	− 1.89 to − 0.13	− 2.37	0.026
GvsI * Control	− 0.98	0.30	− 1.58 to − 0.38	− 3.23	0.002
Marginal R^2^	0.19				
Conditional R^2^	0.59				

Number of Observations = 120. All *p* values in this table are two-tailed. GvsI: GvsI = 1, LvsI = 0. GvsI is the log ratio of global allocation based on self-allocation, and LvsI is the log ratio of local-allocation based on self-allocation. Control: control condition = 1, punishment condition = 0. The intercept estimate represents the allocation’s log-ratio to local in the punishment condition when the reference component is allocation to self. This table provides a summary of the log-ratio analysis. In the punishment condition, allocations to local groups and the global group were higher than in the control condition. There were no significant differences between self and local or between local and global in the punishment condition. Overall, participants exhibited more cooperative behavior under the punishment condition.

**Table 2:** Mixed-model analysis in the control condition.

Random effects	Variance	*Std.Dev*
Individual	0.71	0.84
3-person group	0.37	0.61
6-person group	0.24	0.48
Residual	1.19	1.09

Fixed effects	Estimates	*SE*	*95% CI*	*t*	*P*
Intercept	− 0.73	0.27	− 1.32 to − 0.13	− 2.67	0.02
GvsI	− 0.59	0.20	− 0.99 to − 0.19	− 2.95	0.005
Marginal R^2^	0.03				
Conditional R^2^	0.54				

Number of observations = 60. All *p* values in this table are two-tailed. GvsI: GvsI = 1, LvsI = 0. GvsI is the log-ratio of global allocation based on self-allocation, and LvsI is the log-ratio of local-allocation based on self-allocation. The intercept estimate represents the allocation’s log-ratio to local in the control condition when the reference component is the allocation to self. This table provides a summary of the log-ratio analysis, which was conducted to extract only the control condition. In the control condition, allocations to self were higher than to local groups, and allocations to local groups were higher than to the global group.

**Result 1** Punishment increased cooperation: compared to the control condition, punishment reduced selfish allocations and increased both local and global contributions.

To assess robustness, following previous studies, we conducted complementary analyses on the raw allocation data without compositional transformation using both linear mixed-effects models and ordinary least squares (OLS) regressions. First, using data aggregated at the individual level (N = 120; one observation per participant), we estimated linear mixed-effects regression models with random intercepts at the six-person group level to ensure comparability with the main analyses. These analyses showed that punishment significantly increased global contributions (*b* = 9.09, SE = 3.69, 95% CI [1.35, 16.84], *p* = 0.024) and reduced self-allocation (*b* =  − 10.94, SE = 2.72, 95% CI [− 16.65, − 5.23], *p* = 0.001), while local allocations did not differ significantly between conditions (*b* = 1.84, SE = 2.61, 95% CI [− 3.65, 7.33], *p* = 0.490). These findings remained robust when including control variables (age, gender, seriousness of participation; see Supplementary Tables S1-a and S1-b). Second, to compare with prior studies (e.g., Otten et al.^21^), we estimated OLS regression models based on round-level data (N = 2400 observations; 120 participants × 20 rounds) with standard errors clustered at the individual level to account for repeated observations. The OLS results were qualitatively consistent with those obtained from the mixed-effects models, both with and without control variables (see Supplementary Tables S1-c and S1-d).

Additionally, we examined participants’ net earnings, accounting for both contribution and punishment costs as a supplemental analysis (Supplementary Tables S2 and S3). No significant difference in net earnings was observed between the punishment and control conditions (*p* > 0.10).

### Punishment toward in-group vs. out-group members

**Fig. 4:**
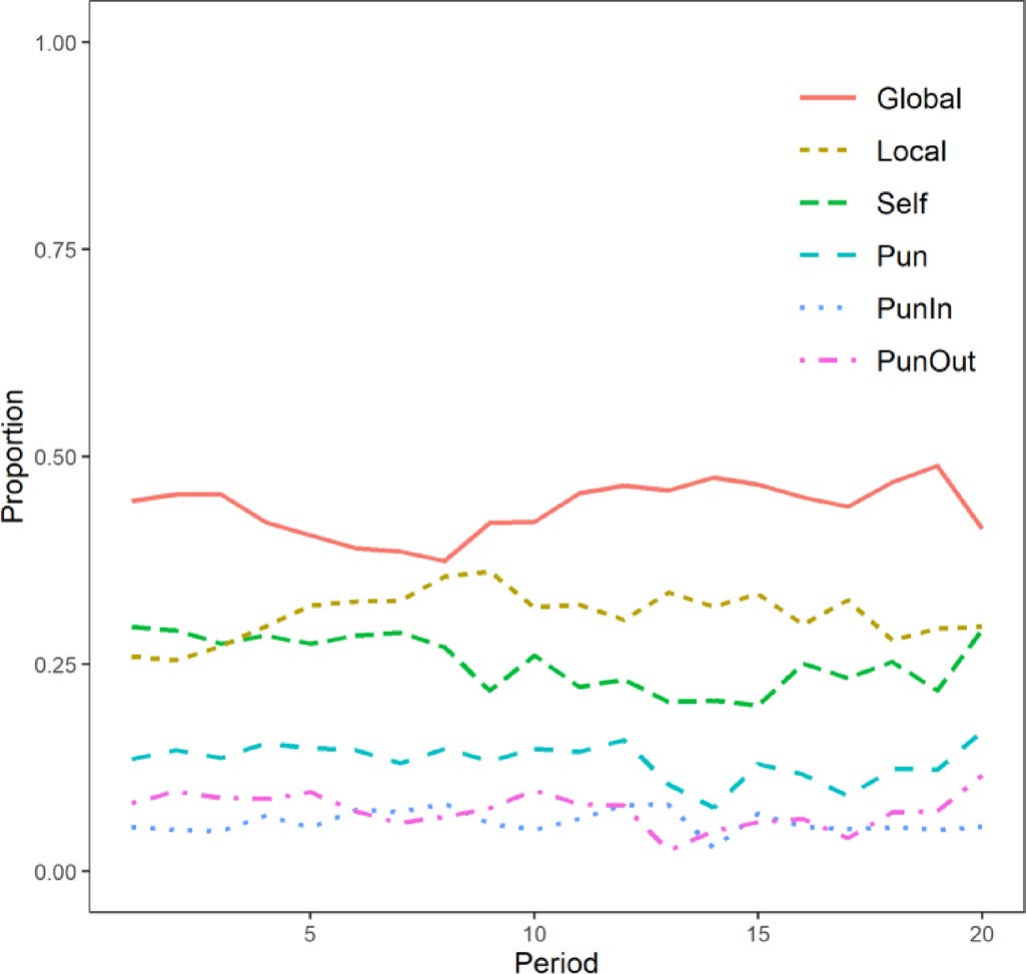
The transition of mean punishment proportion per period. The red solid line (Global) represents allocations to the global public good, the yellow dashed line (Local) represents allocations to the local public good, and the green dashed line (Self) indicates allocations kept for oneself. The blue dashed line (Pun) represents the proportion of points used for punishment, while the light blue dotted line (PunIn) and the pink dash-dotted line (PunOut) represent punishment directed at in-group and out-group members, respectively. Allocations proportions (Global, Local, Self) were calculated as points allocated out of a maximum of 40; punishment proportions (Pun, PunIn, PunOut) were calculated as points assigned out of a maximum of 20. This figure shows the average allocation proportions in the punishment condition over 20 periods.

In MPGG, punishment led to cooperative behavior. Subsequently, to examine the participants’ cooperation norms, we analyzed which cooperative behaviors were punished by which local groups. Punishment behavior was observed consistently across all 20 periods (Fig. 4). The mean punishment expenditure per period by participants was 2.67 points.

Next, we divided punishment into that toward members of the same local group and that toward different local groups, namely in-group and out-group punishment, respectively. Participants punished both in-group and out-group members at comparable levels: the mean total punishment toward other in-group members (excluding oneself) was 1.19 points, whereas that toward out-group members was 1.48 points. Adjusted per potential target, the mean punishment for each in-group and out-group member was 0.59 and 0.49 points, respectively.

A Wilcoxon signed-rank test (two-tailed) showed no significant difference in total punishment between in-group and out-group members (*Md*_in-group_ = 0.25, *Md*_out-group_ = 0.50, *Z* =  − 1.11, *p* = 0.27). After adjusting per target, the difference remained insignificant (*Md*_in-group_ = 0.13, *Md*_out-group_ = 0.17, *Z* = 0.33, *p* = 0.75). Punishment behavior was stable throughout the experiment and was applied similarly to in-group and out-group members, indicating impartial enforcement of cooperative norms.

**Result 2** Punishment did not differ significantly between in-group and out-group members.

### Behavioral targets of punishment

To identify which cooperative behaviors triggered punishment and by whom, we fitted linear mixed models for the total punished amount, in-group punishment, and out-group punishment, with the log-ratios of global and local allocations as fixed effects and 6-person and 3-person groups as random effects (Table 3).

**Table 3:** Linear mixed-model analysis of punished behavior.

	Total punishment		Punishment by in-group		Punishment by out-group	
Random effects	Variance	*Std.Dev*	Variance	*Std.Dev*	Variance	*Std.Dev*
Individual	5.47	2.34	4.48	2.12	0.96	0.98
3-person group	0.93	0.97	0.99	0.99	0.39	0.63
6-person group	2.07	1.44	–	–	1.92	1.39
Residual	20.01	4.47	6.61	2.57	12.87	3.59

Fixed effects	Estimates	*SE*	*95% CI*	*t*	*P*	Estimates	*SE*	*95% CI*	*t*	*p*	Estimates	*SE*	*95% CI*	*t*	*p*
Intercept	3.26	0.60	1.90 to 4.63	5.40	< 0.001	1.44	0.36	0.62 to 2.25	3.98	0.003	1.82	0.49	0.71 to 2.93	3.72	0.005
GvsI	− 0.56	0.08	− 0.71 to − 0.41	− 7.39	< 0.001	− 0.22	0.04	− 0.30 to − 0.13	− 4.88	< 0.001	− 0.34	0.06	− 0.46 to − 0.22	− 5.89	< 0.001
LvsI	− 0.28	0.08	− 0.44 to − 0.12	− 3.42	0.001	− 0.16	0.05	− 0.25 to − 0.06	− 3.28	0.001	− 0.12	0.06	− 0.25 to 0.01	− 1.86	0.067
Marginal R^2^	0.11					0.05					0.07				
Conditional R^2^	0.37					0.48					0.26				

Number of observations = 1200. All *p* values in this table are two-tailed. The GvsI and LvsI estimates represent the log-ratio of the global and local-allocations, respectively, in the punishment condition when the reference component is the allocation to the self. In the punishment by in-group model, the random effect of the 6-person group was excluded from the analysis because it had too little variance for estimation. This table provides a summary of the types of cooperative behavior that were the targets of punishment and were punished by the in/out-group. The total amount of punishment increased as global and local allocations decreased. In-group punishment increased with decreasing allocations to global and local groups, while out-group punishment increased with decreasing global allocation but was not associated with local allocation. Comparatively, global allocation had a greater impact on punishment than local allocation.

For total punishment, both lower global (*b* =  − 0.56, SE = 0.08, 95% CI [− 0.71, − 0.41], *t* =  − 7.39, *p* < 0.001) and lower local (*b* =  − 0.28, SE = 0.08, 95% CI [− 0.44, − 0.12], *t* =  − 3.42, *p* = 0.001) allocations significantly predicted greater punishment levels. For in-group punishment, both global (*b* =  − 0.22, SE = 0.04, 95% CI [− 0.30, − 0.13], *t* =  − 4.88, *p* < 0.001) and local (*b* =  − 0.16, SE = 0.05, 95% CI [− 0.25, − 0.06], *t* =  − 3.28, *p* = 0.001) allocations showed significant negative effects. For out-group punishment, lower global allocation again predicted stronger punishment (*b* =  − 0.34, SE = 0.06, 95% CI [− 0.46, − 0.22], *t* =  − 5.89, *p* < 0.001), while the effect of local allocation was only marginal (*b* =  − 0.12, SE = 0.06, 95% CI [− 0.25, 0.01], *t* =  − 1.86, *p* = 0.067). Across all models, the absolute effect of global allocation was greater than that of local allocation, indicating that insufficient contribution to the global good was the primary target of punishment. Both in-group and out-group members punished free-riding on the global public good, reinforcing collective-level cooperation under punishment.

**Result 3** Non-cooperation, particularly failure to allocate to the global good, was the main target of punishment.

## Discussion

The present study examined three aspects of cooperation and punishment in repetitive MPGG: (i) Can punishment increase cooperation? If yes, does it increase cooperation with a local or global group? (ii) Which among the in-group and out-group receives more punishment? and (iii) What kinds of behavior are punished?

Results showed that punishment induced global cooperation, local cooperation, or both during repeated MPGG sessions. Although some groups achieved a mix of cooperation with global and local groups, other groups achieved either mutual global or local cooperation. This finding suggested that punishment led to specific cooperative norms, upon which members agreed, through repetitive interaction. No significant difference was recorded in the amount of punishment received by in-groups and out-groups. Regardless of whether the target of punishment was an in-group or out-group member, non-cooperation was more likely to be punished, while punishment toward global cooperation was less likely. In-group members were less likely to be punished for cooperating with local groups, whereas punishment of out-group members were neither more nor less likely. These results imply that the norm of punishing non-cooperation, one of the three cooperative norms posited in the introduction, was shared. However, it should be noted that this is only an aggregated tendency and differs from a particular tendency by group.

Although our results indicate that punishment increased cooperation without improving net earnings in the short run, previous research indicates that its welfare effects may differ in the long run. In particular, Gächter et al.^32^ demonstrated that punishment can lead to higher overall efficiency in repeated interactions by sustaining cooperation over time. In line with this perspective, our findings suggest that, while punishment may not enhance welfare immediately, it may yield long-term benefits.

The current study contributes to existing research through the finding that punishment is an institution that induces cooperation in MPGG. Without punishment, non-cooperation gradually increased; this results in mutual non-cooperation in repetitive MPGGs, similarly to standard social dilemmas. This finding is consistent with previous studies on repeated MPGGs^3,4^. However, the effects of the punishment on cooperation were not consistent with Otten et al.^21^. We demonstrated that the sanction in MPGG increases cooperation; the cause may be that those experimental settings regarding the number of group members and repetition made cooperation within or between groups more difficult. In fact, in the present study, participants in the control condition did not contribute more than half to global or local groups. The difficulty of achieving cooperation might make individuals expect to solve the problem through punishment. Punishment can reduce the fear of exploitation and induce the expectation of cooperation.

Another contribution of our study is that it highlights what behaviors are punished in MPGGs, differentiating between in- and out-groups. Our results indicated that, in the MPGG, non-cooperative behavior tended to be punished, regardless of the group. In-group members were less likely to be punished for cooperating with the local group; hence, it is possible that the in-group members tolerated in-group favoritism aimed at maximizing in-group benefits. However, less punishment for global cooperative behavior, irrespective of the group, can be interpreted as subjects’ intention to maximize collective benefits. This finding suggests that, in MPGGs, cross-group cooperation is achievable. While previous studies have provided much evidence of ingroup favoritism, our results suggest that, in the MPGG, participants do not persist in in-group cooperation.

To assess robustness, we compared the results of linear mixed-effects models and OLS regressions on raw allocation values with those obtained from compositional data analysis. The OLS analyses indicated that allocations to the global public good were higher under punishment, while local allocations did not differ significantly between conditions. In contrast, the compositional data analysis suggested that both global and local allocations were higher under punishment. This difference reflects the nature of the data: in raw values, punishment mainly reduced allocations to the self while maintaining local allocations, whereas compositional data analysis detected the relative increase in local allocations when self allocations decreased. Therefore, applying compositional data analysis provided a more precise understanding of how punishment shapes the distribution of limited resources across global, local, and self components.

While our study focused on the outcomes of punishment, the underlying decision-making process has not been directly examined. Further, this aspect has been less explicitly discussed in the MPGG literature. One possible way to conceptualize behavior in this setting is as a two-step decision-making process: first, deciding how much to contribute in total, and second, how to allocate this amount between the local and the global accounts. Previous studies have shown that when global MPCR increases relative to local MPCR, total contributions do not increase and contributions shift from the local to the global account^5,7^. It remains an open question whether punishment primarily affects the first step of increasing overall contributions or whether it also influences the second step through norms and the identification of who is punished. Our conclusions are derived under MPCR_local_  = 2/3 and MPCR_glob_  = 1/2 (with TB_glob_  = 3 > TB_local_  = 2). Different MPCR configurations (e.g., equal MPCRs for local and global) would alter the private–collective trade-off and could shift allocations; exploring such parameter spaces is an important avenue for future work.

A limitation of the current study is that the punishers’ intentions may not be accurately perceived by recipients in our settings. Punishment was anonymous and could be imposed across local groups; thus, recipients could only infer motives from observed allocations. This obscurity of intent may have made it harder for groups to converge on a single cooperative norm, which is consistent with the group-level heterogeneity observed in Fig. 1 (some groups moving toward local cooperation, others toward global cooperation). Future work should examine the boundary conditions—such as punishment transparency and direct measurements of perceived norms—that determine whether groups coordinate on mutual global versus mutual local cooperation.

Another limitation is that the present study may have oversimplified the dynamics in nested group structures in real societies because of laboratory experiments. For example, participants could accurately know all about the behavior of different local groups’ members. In reality, however, information gaps between local groups may be present. Further, lack of information about other groups may affect the use of punishment and make global cooperation difficult. Further studies should incorporate information gaps between groups in experiments to improve external validity.

Our study showed that even in the MPGG setting, non-cooperators tended to be punished, which is consistent with findings from single-level public goods games reported in previous studies^33^. At the same time, Herrmann et al.^33^ emphasized that there is considerable cultural variation in both the targets and the amount of punishment. Because our experiment was conducted in Japan, it remains uncertain whether similar tendencies would be observed in other cultural contexts. Notably, the mean amount of punishment used in our study was 13.35%, which is relatively low compared to previous findings (e.g., Herrmann et al.^33^). This raises the possibility that our results may in part reflect cultural specificities.

Nevertheless, this research showed the positive effect of punishment in increasing cooperation consistent with standard social dilemmas, with a cooperative norm being observed under the punishment system in MPGG. These findings expand the insights into the cooperative behavior of human beings. This research provides clues for understanding cross-group cooperation in multilayered societies and how to work together to tackle existing global cooperation issues.

## Methods

### Participants

The participants’ sample included 120 students from Kansai University (57 men, 63 women) between 18 and 30 years of age (*M* = 20.04, *Std.Dev*. = 1.66 years). All participants received monetary rewards for participation, with a show-up fee of JPY 500 and a mean payoff per participant (including the show-up fee) of JPY 2,141 (range JPY 1,386–3,082; USD 1 = JPY 114). The experiment was conducted on February 21st and 22nd, 2018 and August 6th and 7th, 2018. To check random assignment, we compared the control and punishment conditions on gender, age, and seriousness of participation (7-point item: “How seriously did you participate in the experiment?”). Independent-samples *t*-tests (for continuous variables) and a χ2 test (for gender) showed no significant differences between conditions (all *p* > 0.60; see Supplementary Table S4).

The experimental design included a punishment system (no punishment vs. punishment) as a between-participants design. Participants were randomly allocated to one of the two experimental conditions (control, punishment).

The sample size was determined with reference to the group structure used in Fellner and Lunser^4^. We aimed for 108 participants to maintain balanced conditions and group sizes. Post hoc, a sensitivity power analysis using G*Power^34^ confirmed that this sample size provided 80% power to detect effects at the f = 0.10 level with α = 0.05, exceeding the required sample size of 98. We initially targeted N = 108 to balance groups; the final sample was N = 120.

### Experimental design

To investigate the effect of punishment on global or local cooperation, we conducted a stage game comprising an allocation stage and a punishment stage, introducing a control condition and a punishment condition. The difference between control and punishment conditions was the addition of a second stage with punishment after the simultaneous allocation decision. In the control condition, participants were not given the opportunity to punish anyone during the stage game. In the punishment condition, participants were allowed to punish each other simultaneously at the punishment stage. Administering punishment incurred a cost to the punisher. Participants received an endowment from the experimenter, which they could use to impose punishments. By utilizing this endowment, participants could reduce the earnings of the targeted individual; deducted earnings were not redistributed to any participant, including punishers. If participants chose not to use their punishment endowment, it was retained as part of their final earnings. Thus, exercising punishment decreased the punisher’s payoff; maximizing one’s own payoff required keeping the punishment endowment rather than using it for punishment. The stage game was iterated 20 times.

The stage game began with the allocation stage. Each participant received 40 points at the start of the stage. They decided how to allocate these 40 points to the 6-person group as global cooperation (global), the 3-person group as local cooperation (local), and themselves as defection (self) on their computer screen by entering a value from 0 to 40. The total points that the six members assigned to the global group were summed, then tripled by the experimenter, and equally divided by 6. The total points that the three members assigned to the local group were summed, doubled by the experimenter, and equally divided between the three members of the local group. Allocations by members of the other local group to their own local account did not affect a player’s payoff; only allocations within one’s own local group entered the local public-good return. Participants received one point for assigning each point to themselves. The total points acquired by the participants were the sum of the points obtained from each member’s allocation to both the global and local groups, as well as to themself. This implies MPCR_local_  = 2/3 and MPCR_glob_  = 1/2. Using TB = MPCR × *n*, a one-point contribution generates TB_local_ = 2 (2/3 × 3) versus TB_glob_  = 3 (1/2 × 6). Hence, keeping points maximizes the contributor’s private payoff (= 1), local dominates global in private return (0.67 > 0.50), while global dominates local in TB (3 > 2). All members made this decision simultaneously. Subsequently, participants received feedback on the number of points they allocated to the global group, local groups, and self, along with the points attributed to each allocation and total points earned.

After providing feedback on the allocation stage, the punishment stage followed in the punishment condition. At the start of this stage, participants were given 20 points for punishment and decided the number of points they would deduct to punish each of the five other participants. The point used for punishment was tripled and deducted from the target person’s earnings. After the participants decided to punish, everyone received feedback on the points for which they were punished. Participants were informed that 1 point = 1 yen ($0.01) and that they would receive monetary rewards based on the points earned after completing all periods. Additionally, they were informed that everyone shared the same payoff process.

### Procedure

Before the game began, participants were randomly and anonymously assigned to global groups of size n = 6 or local groups of size n = 3 nested in a given group of 6. The group composition remained the same for the 20 periods. We informed the participants that the decision-making would be repeated 20 times and that they were part of a 6-person group. A minimum of twelve participants participated in each session. None knew who belonged to which group. Each participant was assigned a name from A to F and was informed that A, B, and C were members of one 3-person group, and D, E, and F were members of another 3-person group.

Upon arrival at the laboratory, the participants drew lots to decide on their seats and were seated at computer terminals separated by partitions. All decisions were made using computers and participants could not communicate with each other during the experiment. Upon arrival of all participants, written instructions explaining the game, payoffs, and procedure were read aloud. Subsequently, participants performed practice exercises to confirm their understanding of the payoff structure. When their answers were incorrect, the experimenter explained to them until they answered all questions correctly. When all participants understood the game’s rules, the game began. After the experiment, all participants completed a questionnaire. The average completion time was approximately 60 min in the control condition and 90 min in the punishment condition. Experiments were conducted using a z-Tree^35^.

### Statistical methodologies

#### Compositional data analysis

**Fig. 5:**
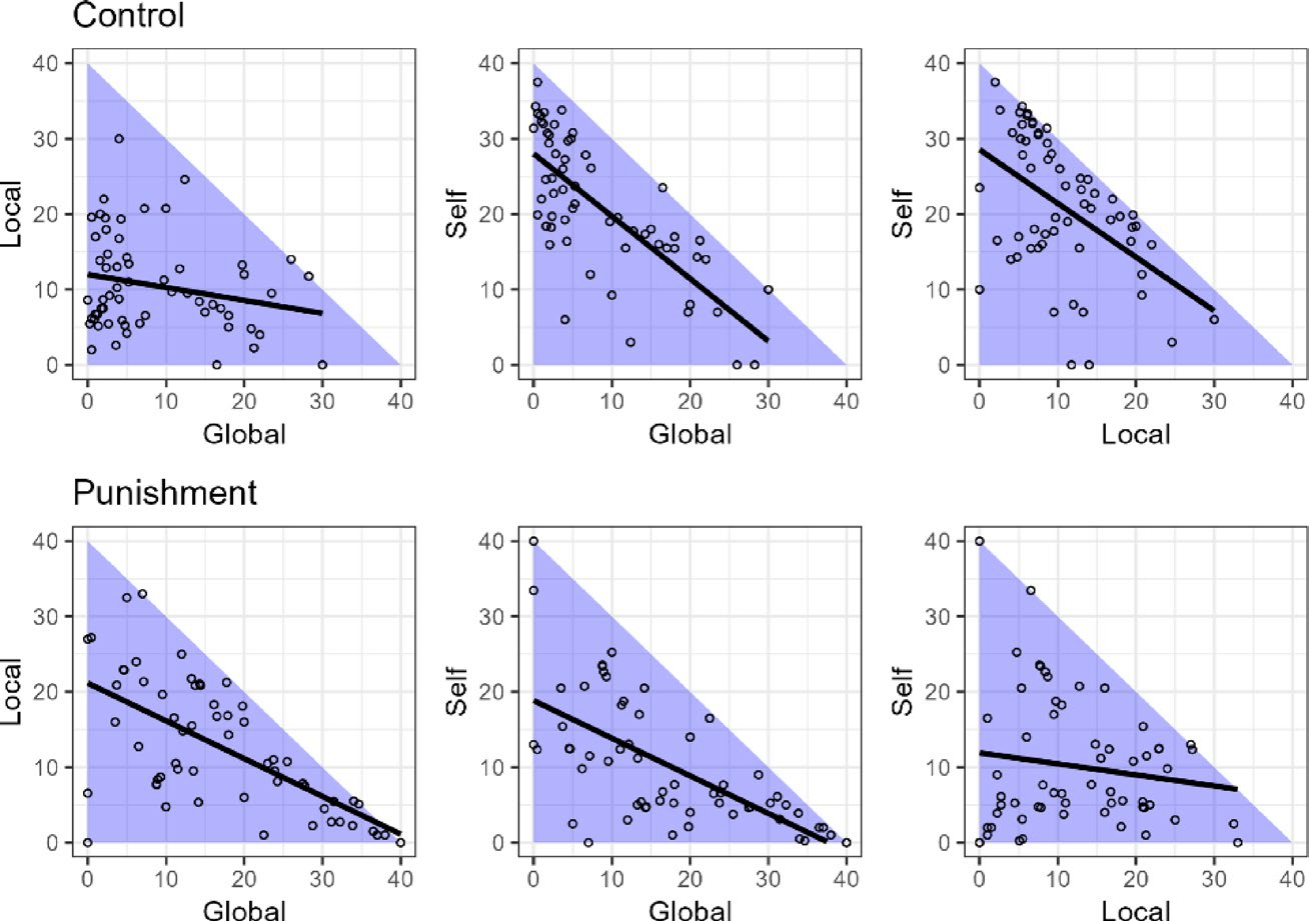
The compositional data bivariable plots of allocation to self, local, and global accounts. The three plots above represent the control condition, and the three plots below display the punishment condition. Circles represent each participant’s data points. The lines represent linear regression. The three plots above represent the control conditions, and the three plots below display the punishment conditions. Circles represent each participant’s data points. The lines represent linear regression. The concentrated distributions and negative correlations observed in these plots arise from the compositional data structure, where allocations to self, local, and global accounts sum to a constant.

Because individuals allocate their resources in three parts (individual, local group, or global group) in MPGG, individuals’ decisions in MPGG cannot be evaluated with one data point only, such as the cooperation rate in standard social dilemmas. Behavioral data in the MPGG must be treated as compositional data in which all variables have positive values and the sum is constant. The independence of the variables cannot be maintained in compositional data; when one variable increases, other variables are forced to decrease, meaning that a general statistical analysis, such as calculating mean values, correlation coefficients, confidence intervals, and multivariate analyses, cannot be performed^36–38^. Our experimental data show a concentrated distribution and a negative correlation arises from the data structure (Fig. 5).

#### Log-ratio analysis

Compositional data is an important format for which analytical methods have been studied. “Log-ratio analysis”^37^ enables us to perform all existing calculations and statistics applied to real numbers. As the sum of the compositional data is constant, the proportion of one component is uniformly determined from the others. Thus, the data can be represented by the number of components − 1 variables using the ratios of variables normalized by a common variable. Although Aitchison^37^ provided several transformation methods, we used the simplest method—the additive log-ratio transformation. The equation defines the following:

$$alr_{i} = \ln \frac{i}{X}.$$

Here, *X* is the reference component and *i* is the component we focused on. Considering that 40 points are allocated as 10, 15, and 15 to global, local, and self-accounts, respectively, and that the reference component is allocation to self, we can calculate the additive log-ratio as follows:

$$alr_{global} = \ln \frac{10}{{15}} = - 0.4054, $$

$$alr_{local} = \ln \frac{15}{{15}} = 0.$$

When the value of the reference component *X* = component *i*, the calculated value is 0 and the larger the value of component *i*, the larger the alr.

Notably, zero replacement must be performed before log-ratio transformation. The log-ratio cannot be calculated if the value of a particular component is zero. Thus, we replaced zero data with a small value among several appropriately submitted methods (e.g., Martín-Fernández, Barceló-Vidal & Pawlowsky-Glahn^30^).

The transformed log-ratio data exhibit several notable features. Log-ratio data follow a multivariate normal distribution and, after normalizing the components, the essential properties of the data remain unchanged. These features are advantageous for statistical analysis, allowing for the application of conventional statistical methods in the log-ratio transformation of compositional data.

### Analysis

We transformed the global allocation and local-allocation data, considering allocation to self as a reference component with additive log-ratio transformation. The transformed values were analyzed using a linear mixed model. We used R (version 4.0.5) for the statistical analysis.

### Transparency and openness

We report how we determined our sample size, all data exclusions (if any), all manipulations, and all measures in the study. The data, code, and materials are publicly accessible at [https://osf.io/4tgam/?view_only=f393271a5d1f46d2a4d6f1e9a41b5ed8]. There is no preregistration for this study.

## Supplementary Information

Below is the link to the electronic supplementary material.

Supplementary Material 1

## Data Availability

The data, code, and materials are publicly accessible at [https://osf.io/4tgam/?view_only=f393271a5d1f46d2a4d6f1e9a41b5ed8].
