# Supplementary material for: The effect of punishment on cooperation in a multilevel public goods game: compositional data analysis
Source: Sci Rep. 2026 Mar 2;16:11552. doi: 10.1038/s41598-026-39950-1 (PMC13056944; doi:10.1038/s41598-026-39950-1)
Supplement: Supplementary file 1 — Supplementary Material 1 [file 41598_2026_39950_MOESM1_ESM.docx]

# Supplementary Information

**Title: The Effect of Punishment on Cooperation in a Multilevel Public Goods Game: Compositional Data Analysis**Authors: Yoko Kitakaji^*^ and Misato Inaba
*Corresponding author: kitakaji@hiroshima-u.ac.jp

## Supplementary Tables

**Supplementary Table S1-a**.

*Linear mixed-effects regression results on raw allocation data (without compositional transformation)*

| Outcome | Marginal R^2^ | Predictor | B | SE | 95% CI | *t* | *p* |
| --- | --- | --- | --- | --- | --- | --- | --- |
| Global contributions | 0.17 | Intercept | 8.42 | 2.61 | 2.94 – 13.89 | 3.23 | 0.005 |
|  |  | Punishment | 9.09 | 3.69 | 1.35 – 16.84 | 2.47 | 0.024 |
| Local contributions | 0.01 | Intercept | 10.55 | 1.85 | 6.66 – 14.43 | 5.71 | <0.001 |
|  |  | Punishment | 1.84 | 2.61 | -3.65 – 7.33 | 0.71 | 0.490 |
| Total contributions | 0.27 | Intercept | 18.96 | 1.92 | 14.93 – 23.00 | 9.87 | <0.001 |
|  |  | Punishment | 10.94 | 2.72 | 5.23 – 16.65 | 4.02 | 0.001 |

*Note. Estimates are based on linear mixed-effects regressions of raw allocation values without compositional transformation. Punishment was coded as 0 = control and 1 = punishment condition. Analyses are based on data aggregated at the individual level, with each participant contributing one observation (N = 120), and models include random intercepts at the 6-person group level. Significance is based on two-tailed tests.*

**Supplementary Table S1-b.**

*Linear mixed-effects regression results on raw allocation data (without compositional transformation) with control variables*

| Outcome | Marginal R^2^ | Predictor | B | SE | 95% CI | *t* | *p* |
| --- | --- | --- | --- | --- | --- | --- | --- |
| Global contributions | 0.21 | Intercept | -1.85 | 9.55 | -20.84 – 17.15 | -0.19 | 0.848 |
|  |  | Punishment | 9.07 | 3.64 | 1.41 – 16.72 | 2.49 | 0.023 |
|  |  | Age | 0.28 | 0.41 | -0.55 – 1.11 | 0.68 | 0.503 |
|  |  | Female | -2.76 | 1.33 | -5.40 – -0.12 | -2.08 | 0.041 |
|  |  | Seriousness | 0.98 | 0.63 | -0.27 – 2.23 | 1.56 | 0.124 |
| Local contributions | 0.04 | Intercept | -0.71 | 8.33 | -17.30 – 15.88 | -0.09 | 0.932 |
|  |  | Punishment | 1.69 | 2.57 | -3.71 – 7.09 | 0.66 | 0.520 |
|  |  | Age | 0.14 | 0.37 | -0.59 – 0.88 | 0.38 | 0.703 |
|  |  | Female | 0.93 | 1.17 | -1.41 – 3.27 | 0.79 | 0.431 |
|  |  | Seriousness | 1.33 | 0.55 | 0.22 – 2.44 | 2.38 | 0.020 |
| Total contributions | 0.35 | Intercept | -3.11 | 10.32 | -23.74 – 17.53 | -0.30 | 0.766 |
|  |  | Punishment | 10.77 | 2.49 | 5.53 – 16.01 | 4.33 | <0.001 |
|  |  | Age | 0.41 | 0.46 | -0.51 – 1.33 | 0.90 | 0.375 |
|  |  | Female | -1.75 | 1.47 | -4.70 – 1.19 | -1.19 | 0.241 |
|  |  | Seriousness | 2.42 | 0.70 | 1.02 – 3.83 | 3.45 | 0.001 |

*Note. Estimates are based on linear mixed-effects regressions of raw allocation values without compositional transformation. Punishment was coded as 0 = control and 1 = punishment condition. Female was coded as 0 = male and 1 = female. Seriousness was measured with the question “How seriously did you participate in the experiment?” on a 7-point scale. Analyses are based on data aggregated at the individual level, with each participant contributing one observation (N = 118), reflecting missing values in the control variables. Models include random intercepts at the 6-person group level. Significance is based on two-tailed tests.*

**Supplementary Table S1-c.**

*OLS regression results on raw allocation data (individual-level, non-compositional)*

| Outcome | R^2^ | Predictor | B | SE | 95% CI | *t* | *p* |
| --- | --- | --- | --- | --- | --- | --- | --- |
| Global contributions | 0.10 | Intercept | 8.42 | 1.05 | 6.35 – 10.49 | 7.99 | < 0.001 |
|  |  | Punishment | 9.10 | 1.80 | 6.35 – 12.62 | 5.05 | < 0.001 |
| Local contributions | 0.01 | Intercept | 10.55 | 0.81 | 8.95 – 12.14 | 12.95 | <0.001 |
|  |  | Punishment | 1.84 | 1.38 | -0.87 – 4.55 | 1.33 | 0.183 |
| Total contributions | 0.15 | Intercept | 18.96 | 1.18 | 16.65 – 21.28 | 16.07 | <0.001 |
|  |  | Punishment | 10.94 | 1.62 | 7.75 – 14.12 | 6.73 | <0.001 |

*Note. Estimates are based on OLS regressions of raw allocation values without compositional transformation. Punishment was coded as 0 = control and 1 = punishment condition. Standard errors are clustered at the individual level to account for repeated observations across rounds. The number of observations is 2,400 (participants × rounds). Significance is based on two-tailed tests.*

**Supplementary Table S1-d.**

*OLS regression results on raw allocation data (individual-level, non-compositional) with control variables*

| Outcome | R^2^ | Predictor | B | SE | 95% CI | *t* | *p* |
| --- | --- | --- | --- | --- | --- | --- | --- |
| Global contributions | 0.12 | Intercept | -3.12 | 12.46 | -27.55 – 21.32 | -0.25 | 0.803 |
|  |  | Punishment | 9.27 | 1.76 | 5.81 – 12.73 | 5.26 | < 0.001 |
|  |  | Age | 0.21 | 0.58 | -0.93 – 1.34 | 0.36 | 0.722 |
|  |  | Female | -1.94 | 1.91 | -5.69 – 1.81 | -1.01 | 0.310 |
|  |  | Seriousness | 1.34 | 0.83 | -0.28 – 2.97 | 1.62 | 0.106 |
| Local contributions | 0.02 | Intercept | -3.35 | 9.61 | -22.19 – 15.49 | -0.35 | 0.728 |
|  |  | Punishment | 1.52 | 1.35 | -1.13 – 4.17 | 1.13 | 0.260 |
|  |  | Age | 0.21 | 0.42 | -0.61 – 1.03 | 0.51 | 0.611 |
|  |  | Female | 0.30 | 1.55 | -2.75 – 3.34 | 0.19 | 0.849 |
|  |  | Seriousness | 1.60 | 0.59 | 0.44 – 2.75 | 2.7 | 0.007 |
| Total contributions | 0.20 | Intercept | -6.46 | 11.6 | -29.20 – 16.28 | -0.56 | 0.577 |
|  |  | Punishment | 10.79 | 1.53 | 7.75 – 14.12 | 7.06 | < 0.001 |
|  |  | Age | 0.42 | 0.51 | -0.57 – 1.41 | 0.83 | 0.408 |
|  |  | Female | -1.65 | 1.66 | -4.91 – 1.62 | -0.99 | 0.322 |
|  |  | Seriousness | 2.94 | 0.86 | 1.26 – 4.62 | 3.44 | < 0.001 |

*Note. Estimates are based on OLS regressions of raw allocation values without compositional transformation. Punishment was coded as 0 = control and 1 = punishment condition. Female was coded as 0 = male and 1 = female. Seriousness was measured with the question “How seriously did you participate in the experiment?” on a 7-point scale. Standard errors are clustered at the individual level to account for repeated observations across rounds. The number of observations is 2,360 (participants × rounds), reflecting missing values in the control variables. Significance is based on two-tailed tests.*

**Supplementary Table S2.**

*Means and standard deviations of net earnings (excluding punishment endowment and show-up fee)*

|  | Mean | SD |
| --- | --- | --- |
| Control | 1348 | 258 |
| Punishment | 1535 | 312 |

*Note. Net earnings were calculated by subtracting the punishment endowment (20 points × 20 periods = 400 points) The show-up fee was not included in these calculations.*

**Supplementary Table S3.**

*Mixed-model analysis of net earnings (excluding punishment endowment and show-up fee)*

| Random Effects | Variance | *Std.Dev*. |  |  |  |
| --- | --- | --- | --- | --- | --- |
| 3-person group | 2509 | 50.09 |  |  |  |
| 6-person group | 65761 | 256.44 |  |  |  |
| Residual | 19228 | 138.66 |  |  |  |
| Fixed Effects | Estimates | *SE* | *95% CI* | *t* | *P* |
| Intercept | 1347.65 | 83.8 | 1171.60 – 1523.70 | 16.082 | **<0.001** |
| Punishment | 187.65 | 118.5 | -61.33 – 436.62 | 1.583 | 0.131 |
| Marginal R^2^ | 0.092 |  |  |  |  |
| Conditional R^2^ | 0.800 |  |  |  |  |

*Note. Number of observations = 120. Net earnings were calculated by subtracting the punishment endowment (20 points × 20 periods = 400 points) from total payoffs. The show-up fee was not included. All p values are two-tailed.*

**Supplementary Table S4.**

*Randomization Check for Demographic and Post-experiment Variables*

| **Variable** | **Control M (SD)** | **Punishment M (SD)** | **Test statistic** | ***p*-value** |
| --- | --- | --- | --- | --- |
| Seriousness (1-7) | 6.07 (1.04) | 6.05 (0.95) | *t* = 0.09 | .927 |
| Age (years) | 20.0 (1.40) | 20.1 (1.89) | *t* = -0.50 | .619 |
| Gender (% male) | 46.0% | 49.0% | *χ*² = 0.14 | .712 |

*Note. Seriousness was measured with the question “How seriously did you participate in the experiment?” on a 7-point scale. No significant differences were observed between conditions, indicating successful randomization.*
